# The OsmiRNA166b‐OsHox32 pair regulates mechanical strength of rice plants by modulating cell wall biosynthesis

**DOI:** 10.1111/pbi.13565

**Published:** 2021-03-05

**Authors:** Hong Chen, Ruiqiu Fang, Rufang Deng, Jianxiong Li

**Affiliations:** ^1^ Key Laboratory of South China Agricultural Plant Molecular Analysis and Genetic Improvement, and Guangdong Provincial Key Laboratory of Applied Botany South China Botanical Garden Chinese Academy of Sciences Guangzhou China; ^2^ University of Chinese Academy of Sciences Beijing China; ^3^ Institute of Maize and Featured Upland Crops Zhejiang Academy of Agricultural Sciences Dongyang Zhejiang China; ^4^ Public Laboratory of Sciences South China Botanical Garden Chinese Academy of Sciences Guangzhou China; ^5^ State Key Laboratory for Conservation and Utilization of Subtropical Agro‐Bioresources College of Agriculture Guangxi University Nanning China

**Keywords:** cell wall, cellulose, Hox32, lignin, mechanical strength, miR166b, rice

## Abstract

The plant cell wall provides mechanical strength to support plant growth and development and to determine plant architecture. Cellulose and mixed‐linkage glucan (MLG) present in primary cell wall, whereas cellulose, lignin and hemicellulose exist in secondary cell wall. Biosynthesis of the cell wall biopolymers needs the coordinated transcriptional regulation of all the biosynthetic genes. The module of OsmiR166b‐*OsHox32* regulates expression levels of the genes related to biosynthesis of MLG, cellulose and lignin. Transgenic plants knocking down miR166b (STTM166b) by short tandem target mimic (STTM) technology or overexpressing *OsHox32* (OEHox32) showed drooping leaves and brittle culms. Due to accumulation of less lignin and cellulose, the cell wall thickness of STTM166b and OEHox32 plants was reduced when compared to that of wild‐type plants. Overexpression of miR166b (OE166b) in rice plants or knocking down of *OsHox32* by RNA interference (RNAiHox32) led to increased thickness of cell walls and enhanced mechanical strength of culms. Molecular analyses showed that OsmiR166b‐*OsHox32* pair regulates cell wall‐related gene expression. OsHox32 binds to the promoters of *OsCAD2* and *OsCESA7* to suppress the expression levels of these two genes. The suppression of *OsCAD2* is synergistic when *OsHox32* is co‐expressed with *OSH15* (*Oryza sativa* homeobox 15). OsHox32 interacts with OSH15, and the START domain of OsHox32, harbouring the miR166b cleavage site, is required for the interaction of these two proteins. Our results demonstrate that OsmiR166b‐*OsHox32* pair plays important roles not only in plant growth and development but also in plant architecture by regulating the cell wall‐related gene expression.

## Introduction

The plant cell wall is a complex and heterogeneous matrix of polysaccharides, glycoproteins, solutes and enzymes, which governs the morphology, growth and development of plants. Primary cell wall is a polymeric network of crystalline cellulose microfibrils, pectic polysaccharides and xyloglucans, which possesses a remarkable combination of strength and pliancy that enable cells to withstand mechanical forces and to create space for growth (Cosgrove and Jarvis, 2012). Secondary cell wall forms a thick layer rich in cellulose, hemicellulose and lignin to provide mechanical strength and rigidity allowing vascular plants reach great heights and compete for light (Taylor‐Teeples *et al.,* 2015). Synthesis of these cell wall‐related components requires expression of sets of specific genes that are regulated by multiple tiers of transcription factors. Cellulose is synthesized by rosette complexes that consist of multiple cellulose synthase (CESA) proteins. In rice, CESA1, 3 and 8 and CESA4, 7 and 9 are required for cellulose biosynthesis during primary and secondary cell wall formation, respectively (Tanaka *et al.,* 2003; Wang *et al.,* 2010). Lignin biosynthesis is controlled by a set of genes. For example, *PAL1* (phenylalanine ammonia lyase 1), *C4H* (cinnamate 4‐hydroxylase) and *4CL3* (4‐hydroxycinnamate: CoA ligase 3) are responsible for the first three steps of lignin biosynthesis, while *COMT* (caffeic acid 3‐*O*‐methyltransferase) and *CAD* (cinnamyl alcohol dehydrogenase) catalyse the last two steps of biosynthesis (Zhao and Dixon, 2011). Plant growth and development need biosynthesis of the cell wall components that are controlled by different sets of biosynthetic genes, which are in turn controlled by the master switches of NAC domain and homeodomain‐leucine zipper Class III (HD‐ZIP III) transcription factors (Bhargava *et al.,* 2010; Kubo *et al.,* 2005).

Leaf architecture, including leaf shape and leaf angle, is an important agronomic trait that contributes largely to yield by affecting photosynthesis rate and planting density. Moderate width and erectness of leaves make rice plants have an ideal canopy which is the long sought target in rice breeding (Peng *et al.,* 1999). As the importance of rice leaf architecture, scientists have paid much attention to the molecular mechanisms of leaf shape and leaf angle formation, and thus, many genes related to leaf architecture have been identified. The NAC domain transcription factors, such as VASCULAR‐RELATED NAC‐DOMAIN 7 (VND7), SECONDARY WALL‐ASSOCIATED NAC DOMAIN PROTEIN 1 (SND1) and NAC SECONDARY WALL THICKENING PROMOTING FACTOR 1 (NST1), act as master regulators of the secondary cell wall biosynthesis (Endo *et al*., 2015). The Arabidopsis *snd1 nst1* double‐mutant showed pendent inflorescence stem and reduced stem strength due to the defective deposition of cellulose and lignin (Zhong *et al.,* 2011). Rice transcription factors SECONDARY WALL NAC DOMAIN PROTEINs (OsSWNs) regulate secondary cell wall formation, and repression of *OsSWN*s expression in rice resulted in drooping leaves and reduced plant height (Yoshida *et al.,* 2013).

Culm stiffness is related to lodging resistance that plays important roles in grain yield and quality. Several factors such as the thickness and the chemical contents of cell walls have been reported to affect culm stiffness. Different genetic approaches have been applied to improve rice lodging, for example, the semidwarf gene *sd‐1* has been used to reduce plant height and relatively increase sturdiness of the basal culms leading to the improvement of lodging (Sasaki *et al.,* 2002). In general, lignin and/or cellulose determine physical strength of culms, thus reduced contents of lignin and/or cellulose lead to brittle culms (Li *et al*., 2003; Vega‐Sánchez *et al.,* 2012). The maize *brown midrib 1* (*bm1*) mutant, harbouring a mutation in the lignin biosynthetic *CAD* gene, shows a substantial reduction in the total lignin content (Halpin *et al.,* 1998). The rice *flexible culm 1* (*fc1*) mutant was identified as disrupting the *CAD7* gene by a T‐DNA insertion, and the mutation caused a reduction in cell wall thickness and a decrease in lignin content, leading to reduced mechanical rigidity of culms (Li *et al.,* 2009). Rice *GOLD HULL AND INTERNODE 2* (*GH2*) gene encodes CAD2, and the *gh2* mutant is lignin‐deficient and shows reduced lignin content in cell walls (Zhang *et al.,* 2006).

The Arabidopsis KNOX protein BP (BREVIPEDICELLUS) regulates lignin content by suppressing the expression of a set of lignin biosynthetic genes, including *At4CL1* (4‐coumarate: CoA ligase 1), *AtC4H*, *PAL1*, *CCoAOMT* (caffeoyl‐CoA *O*‐methyltransferase) and *COMT1*. EMSA (Electrophoretic mobility shift assay) demonstrated that BP binds to the promoters of *COMT1* and *CCoAOMT* and suppresses the expression of these two genes (Mele *et al.,* 2003). A rice KNOX gene *Oryza sativa homeobox 15* (*OSH15*) is highly expressed in the abscission zone and OSH15 protein suppresses the expression of *CAD2* by binding to its promoter, which leads to reduced lignin accumulation in the abscission zone and consequentially causes grain shattering (Yoon *et al.,* 2017).

Homeodomain‐leucine zipper (HD‐ZIP) transcription factor family is unique to the plant kingdom and classified into four groups (I–IV) (Elhiti and Stasolla, 2009). It has been reported that expression of HD‐ZIP III genes is regulated by miR165/166 in Arabidopsis and by miR166 in rice at the posttranscriptional level (Rhoades *et al.,* 2002; Zhang *et al.,* 2018). Short Tandem Target Mimic (STTM) technology has been used to study the function of microRNAs (Gao *et al.,* 2018; Yan *et al.,* 2012). Our previous study showed that OsmiR166b is predicted from a QTL region contributing to grain yield of a heterotic rice hybrid (Fang *et al.,* 2013; Li *et al.,* 2000), and however, the function of OsmiR166b in rice growth and development is still elusive. In this study, we studied the function of OsmiR166b and its predicted target gene *OsHox32*, a member of the HD‐ZIP III group, in plant architecture formation. The OsmiR166b‐*OsHox32* module affects leaf shape and culm rigidity by regulating expression levels of the cell wall‐related genes, leading to altered thickness of cell walls and mechanical strength of plants. The transcription factor OsHox32 suppresses the expression of *OsCAD2* and *OsCESA7* by binding to their respective promoter, thus leading to reduced lignin and cellulose contents.

## Results

### STTM166b and OEHox32 plants show drooping leaves and brittle culms

OsmiR166b has been shown to target genes encoding START domain‐containing proteins (http://structuralbiology.cau.edu.cn/PNRD/). To further study the function of OsmiR166b in rice, we selected *OsHox32* (Loc_Os03g43930), one of the START domain‐containing genes, as the candidate target to pair with OsmiR166b (Figure S1a). To confirm that *OsHox32* is an authentic target of OsmiR166b, we randomly mutated the nucleotides in the OsmiR166b‐cleaving site of *OsHox32* and performed RT‐PCR analysis. The results confirmed that OsmiR166b cleaves *OsHox32*, but not the mutated *mOsHox32* (Figure S1b). We generated transgenic plants either knocking down OsmiR166b (STTM166b) by STTM technology or overexpressing OsmiR166b (OE166b) by overexpressing *OsMIR166b*. Meanwhile, we also produced transgenic plants overexpressing or suppressing *OsHox32*. Expression levels of the manipulated genes in the homozygous lines were analysed by qRT‐PCR, and two representative lines for each gene transformation were investigated (Figure S2a, b).

**Figure 1:**
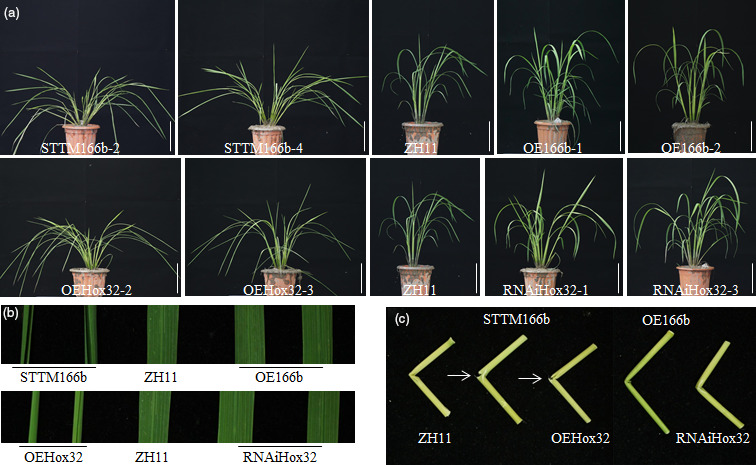
Morphology of transgenic plants. (a) Morphology of wild type (ZH11) and OsmiR166b and *OsHox32* transgenic plants at tillering stage. Bars = 10 cm. (b) Flag leaves from wild‐type and transgenic plants. (c) Breaking points of the culms from ZH11 and transgenic rice plants. Arrows indicate the breaking points.

When grown in the paddy field, STTM166b and *OsHox32* overexpression (OEHox32) plants displayed similar phenotypes, whereas OE166b transgenic plants phenocopied *OsHox32* suppression (RNAiHox32) plants (Figure 1a). Detailed investigation showed that transgenic plants displayed multiple phenotypic alterations in comparison to wild‐type plant ZH11. Although OE166b and RNAiHox32 transgenic plants did not show significant difference in plant height, STTM166b and OEHox32 showed reduced plant height when compared to wild type (WT) (Figure S2c, d). When compared the different internodes of transgenic plants, the first and the second internodes of STTM166b and OEHox32 were significantly shorter than those of ZH11 (Figure S3a, b). Leaves of STTM166b and OEHox32 were adaxially rolled and drooping although those of OE166b and RNAiHox32 did not show difference in comparison with the leaves of ZH11 (Figure 1b, Figure S3c, d). Another prominent feature of STTM166b and OEHox32 plants is the brittle culm that can be broken easily by bending (Figure 1c). Other agronomic traits of the transgenic plants were investigated and listed in Table S1. These phenotypic alterations imply multiple functions of OsmiR166b and *OsHox32* in plant growth and development.

### OsmiR166b‐*OsHox32* pair regulates cell wall thickness

**Figure 2:**
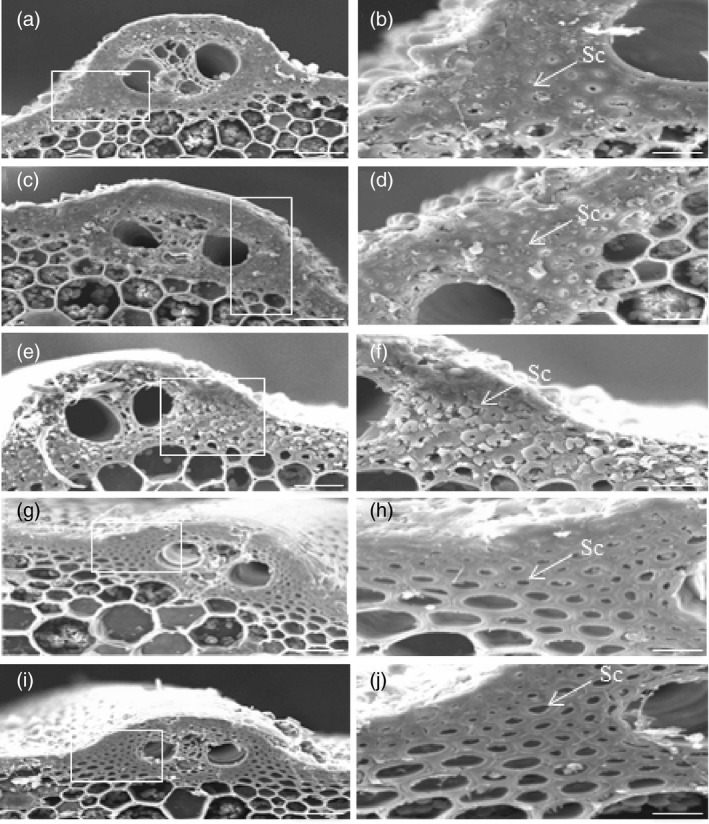
SEM micrographs of the transverse sections of culms showing the state of epidermis and the sclerenchyma cells around the vascular bundle. ZH11 (a, b), OE166b (c, d), RNAiHox32 (e, f), STTM166b (g, h) and OEHox32 (i, j). The white rectangles indicate the parts being magnified in the right panels. Bars = 25 μm (a, c, e, g, i) and 10 μm (b, d, f, g, j). Sc, Sclerenchyma cells.

**Figure 3:**
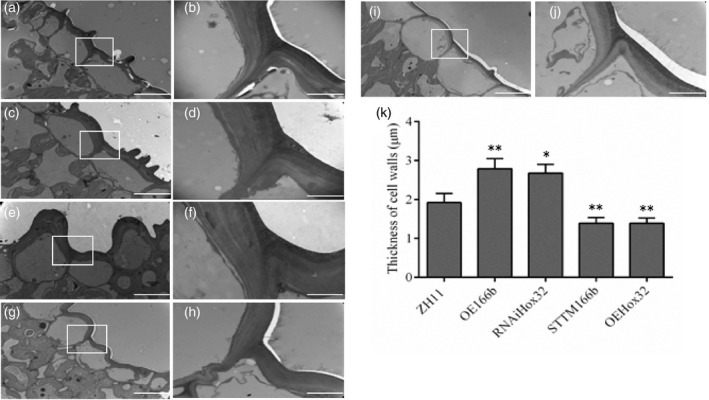
TEM micrographs of the transverse sections of flag leaves showing cell walls of the sclerenchyma cells. ZH11 (a, b), OE166b (c, d), RNAiHox32 (e, f), STTM166b (g, h) and OEHox32 (i, j). The white rectangles indicate the parts being magnified in the right panels. Bars = 10 μm (a, c, e, g, i) and 2 μm (b, d, f, h, j). (k) Statistical analysis of the thickness of cell walls. Data are shown as mean ± SE (*n* = 10), Student’s *t*‐test (two tailed): *, *P* < 0.05; **, *P* < 0.01.

Reduction in the mechanical strength of culms reflects changes in structure and/or composition of the cell walls. In rice plants, cells that are under the epidermis and around the peripheral vascular tissues provide mechanical support for plants. To investigate cell wall morphology, we used scanning electron microscopy (SEM) and transmission electron microscopy (TEM) to examine stem and leaf cells. SEM observation of the culm sections revealed that sclerenchyma cells of ZH11, OE166b and RNAiHox32 plants were almost completely filled up (Figure 2a–f), whereas those cells of STTM166b and OEHox32 culms contained cavities (Figure 2g–j). Furthermore, TEM observation of the leaf sections showed that cell walls of the sclerenchyma cells under epidermis of OE166b and RNAiHox32 plants were significantly thicker than those of ZH11 (Figure 3a–f), whereas cell walls of STTM166b and OEHox32 plants were significantly thinner than those of ZH11 (Figure 3g–j). Statistical data of the cell wall thickness were shown in Figure 3k. These findings suggest that the OsmiR166b‐*OsHox32* pair plays important roles in modulating cell wall thickness, which determines the mechanical strength of plants.

### OsmiR166b‐*OsHox32* pair manipulates cellulose and lignin contents

**Figure 4:**
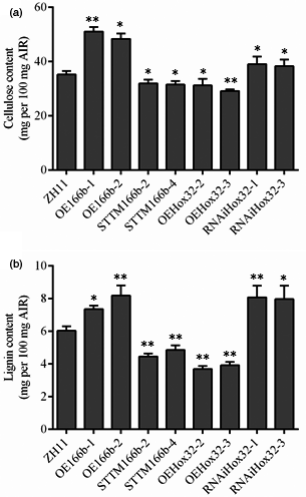
Cellulose and lignin contents in the flag leaves of wild‐type and transgenic plants at tillering stage. Cellulose (a) and lignin (b) contents of flag leaves. Data from five biological repeats are shown as mean ± SE. Student’s *t*‐test. *, *P* < 0.05; **, *P* < 0.01.

**Figure 5:**
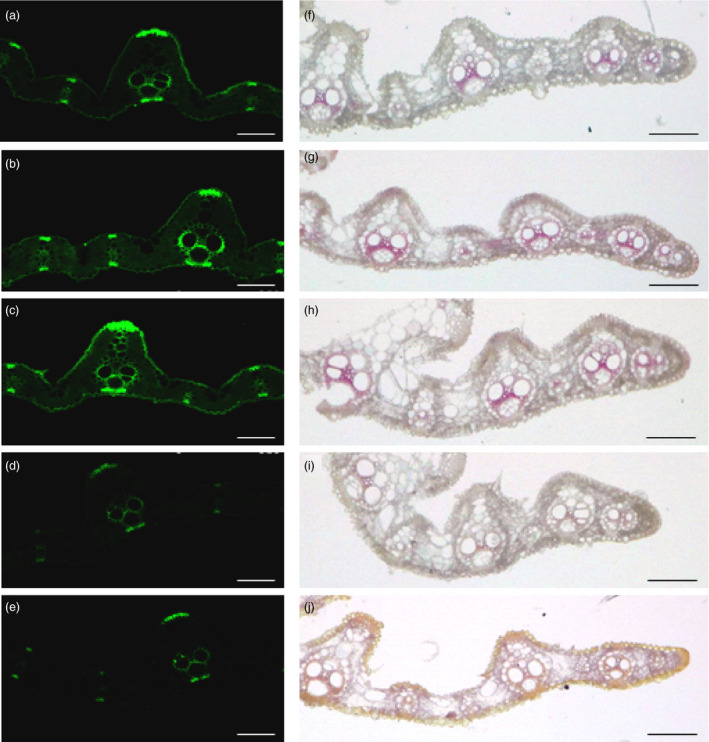
The flag leaf sections stained with Calcofluor White Stain and Wiesner histochemical reagent, respectively, showing cellulose and lignin deposition. (a‐e) Calcofluor White staining exhibits various intensities of green colour proportionally reflecting cellulose deposition in the cells around vascular bundles of ZH11 (a), OE166b (b), RNAiHox32 (c), STTM166b (d) and OEHox32 (e). (f–j) Wiesner histochemical reagent staining shows different coloration in the cells around vascular bundles of ZH11 (f), OE166b (g), RNAiHox32 (h), STTM166b (i) and OEHox32 (j), indicating different lignin deposition. Bars: 100 µm.

The thickness of cell wall is significantly altered in OsmiR166b and *OsHox32* transgenic plants. Cellulose, lignin and hemicellulose are three main components of the cell walls, and therefore, we analysed the contents of these compounds in transgenic plants. Contents of hemicellulose in the leaves were not significantly different between the transgenic and wild‐type plants (Figure S4). We then checked the contents of cellulose and lignin in transgenic plants. Cellulose was significantly reduced in the leaves of STTM166b and OEHox32 plants but significantly increased in OE166b and RNAiHox32 plants when compared with that in ZH11 (Figure 4a). Similar results were observed for lignin in the leaves of OsmiR166b and OsHox32 transgenic plants (Figure 4b). To further investigate the deposition of cellulose and lignin in cell walls, we stained transverse sections of leaves from transgenic and wild‐type plants with Calcofluor White Stain and conventional Wiesner histochemical reagent, respectively. Calcofluor White Stain reagent stains cellulose, and the fluorescent signal reflects cellulose content; on the other hand, conventional Wiesner (phloroglucinol‐HCl) reagent is known to react with cinnamaldehyde residues in lignin, and the colour intensity of staining is an approximate indicator of lignin content. As shown in Figure 5, OE166b and RNAiHox32 exhibited stronger fluorescence whereas STTM166b and OEHox32 exhibited weaker fluorescence when compared with the fluorescence observed in ZH11 (Figure 5a–e), indicating different amounts of the cellulose deposited in the cell walls of transgenic plants. Ectopic staining of lignin was also observed in the leaf transverse sections (Figure 5f–j), and the different colour intensities shown in the vascular bundle cells indicated differential lignin deposition in the secondary walls that more in OE166b and RNAiHox32 plants whereas less in STTM166b and OEHox32 plants when compared with that in ZH11.

**Figure 6:**
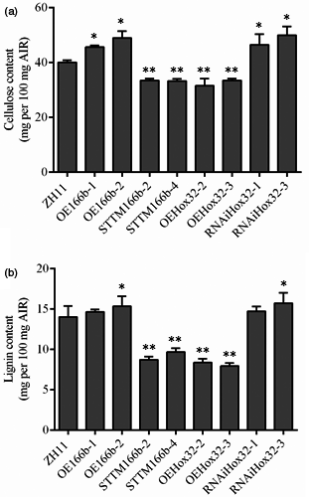
Cellulose and lignin contents in the culms of wild‐type and transgenic plants at heading stage. Cellulose (a) and lignin (b) contents of the second culms counting from the top of rice plants. Data from five biological repeats are shown as mean ± SE. Student’s *t*‐test. *, *P* < 0.05; **, *P* < 0.01.

**Figure 7:**
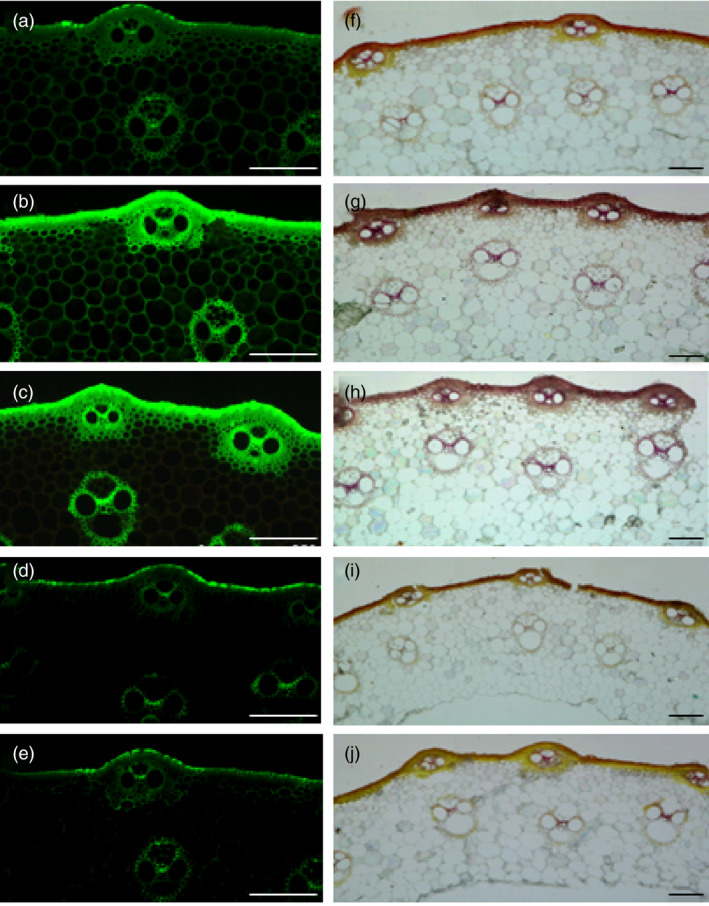
The sections of culms stained by Calcofluor White Stain and Wiesner histochemical reagent, respectively, showing cellulose and lignin deposition. (a–e) Calcofluor White staining of the transverse sections of the second culms shows different intensities of green colour in epidermis and vascular bundles of ZH11 (a), OE166b (b), RNAiHox32 (c), STTM166b (d) and OEHox32 (e), indicating different cellulose deposition in the cell walls. (f–j) Wiesner’s staining of the transverse sections of the second culms exhibits various coloration in epidermis and vascular bundles of ZH11 (f), OE166b (g), RNAiHox32 (h), STTM166b (i) and OEHox32 (j), showing different lignin deposition in the cell walls. Bars: 100 µm.

Furthermore, we also examined the contents of cellulose and lignin in the culms of transgenic plants. As shown in Figure 6, the contents of cellulose and lignin were significantly increased in OE166b and RNAiHox32 plants but reduced in STTM166b and OEHox32 transgenic plants when compared to those of the wild‐type ZH11. In addition, staining of the transverse sections of culms showed that OE166b and RNAiHox32 transgenic plants accumulated more cellulose and lignin, when compared to the wild‐type ZH11, whereas STTM166b and OEHox32 plants had less cellulose and lignin (Figure 7). These results indicate that the OsmiR166b‐*OsHox32* pair regulates the abundance of cellulose and lignin.

**Figure 8:**
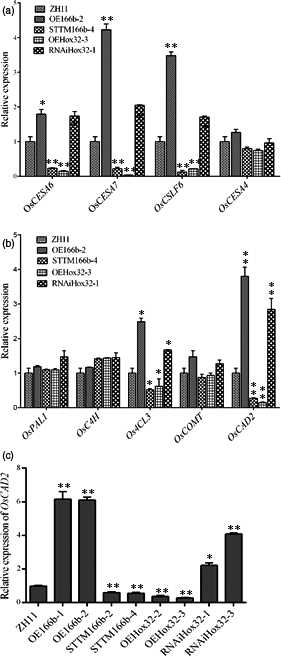
Expression of the cell wall‐related genes. (a) Expression of cellulose and MLG biosynthetic genes in wild‐type and transgenic plants. (b) Expression of lignin biosynthetic genes. (c) Expression of *OsCAD2* in different transgenic plants. *, *P* < 0.05; **, *P* < 0.01.

**Figure 9:**
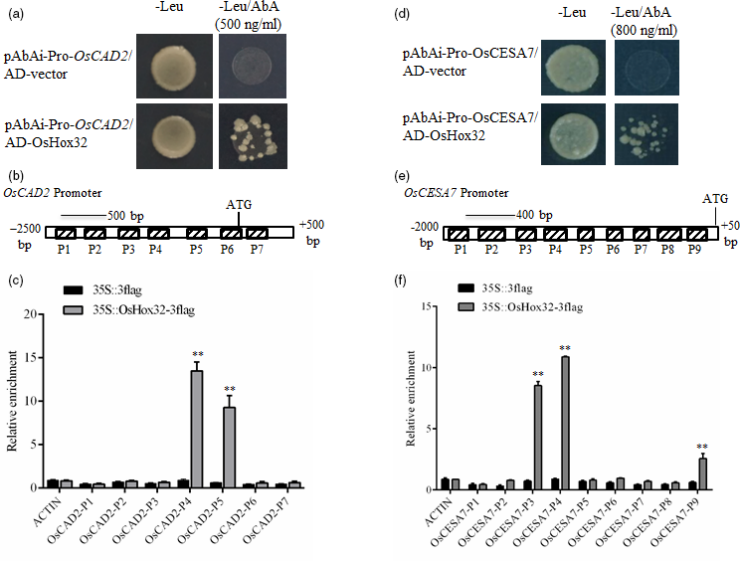
OsHox32 suppresses the expression of *OsCAD2* and *OsCESA7*. (a, d) Y1H assays show OsHox32 binds to the promoters of *OsCAD2* (a) and *OsCESA7* (d). (b, e) Schematic graphs of the promoter and partial coding region of *OsCAD2* (b) and the promoter region of *OsCESA7* (e), and the dashed boxes indicate the binding parts tested in ChIP‐PCR. (c, f) ChIP‐PCR confirmed OsHox32 binds to the P4 and P5 parts of *OsCAD2* promoter (c) and to the P3, P4 and P9 parts of *OsCESA7* promoter (f). The genomic fragment of *Actin 1* gene was used as a negative control. Three biological repeats were performed. Student’ *t*‐test. **, *P* < 0.01.

Biosynthesis of cellulose and lignin was controlled by different sets of genes. Expression levels of several genes involved in cellulose and lignin biosynthesis were examined in the wild‐type and transgenic plants (Figure 8). Quantitative RT‐PCR analyses showed that the transcript levels of the cellulose biosynthetic genes *OsCESA6* and *OsCESA7* and the MLG biosynthetic gene *OsCSLF6* (cellulose synthase‐like F6, a major isoform of the ß‐1,3:1,4‐glucan synthase gene) were significantly increased in OE166b and RNAiHox32 plants but decreased in STTM166b and OEHox32 plants when compared with their expression levels in wild‐type ZH11 (Figure 8a). When checked the expression of lignin biosynthesis genes, we found that the expression levels of *Os4CL3* and *OsCAD2* were significantly increased in OE166b and RNAiHox32 plants but decreased in STTM166b and OEHox32 plants when compared with their expression levels in the wild‐type ZH11. On the other hand, the expression levels of *OsPAL1*, *OsC4H* and *OsCOMT* did not show significant differences between the transgenic and wild‐type plants (Figure 8b). CAD genes function in the last step of monolignol biosynthesis, catalysing hydroxyl‐cinnamyl aldehydes into their corresponding alcohols, and *OsCAD2* is regarded as the *bona fide* CAD gene playing the major role in monolignol biosynthesis (Hirano *et al.,* 2012). The expression of *OsCAD2* was further examined in more transgenic lines, and the results showed that the expression of *OsCAD2* was significantly regulated in OsmiR166b and OsHox32 transgenic plants (Figure 8c). As a transcription factor, OsHox32 may directly regulate the expression of *OsCAD2*. Yeast one‐hybrid (Y1H) assay showed that OsHox32 binds to the promoter region of *OsCAD2* (Figure 9a). To further confirm the result of Y1H assay, we performed ChIP‐PCR analysis. The results showed the P4 and P5 parts of the promoter were enriched by Flag antibody (Figure 9b, c), indicating that OsHox32 binds to these parts of the *OsCAD2* promoter.

*OsCESA7* is an essential gene for biosynthesis of the cellulose in the secondary cell walls. Yeast one‐hybrid assay showed that OsHox32 binds to the promoter region of *OsCESA7* (Figure 9d), and ChIP‐PCR assay confirmed that OsHox32 binds to the P3, P4 and P9 parts of the *OsCESA7* promoter (Figure 9e, f).

### OsHox32 interacts with OSH15 and co‐suppresses the expression of *OsCAD2*

**Figure 10:**
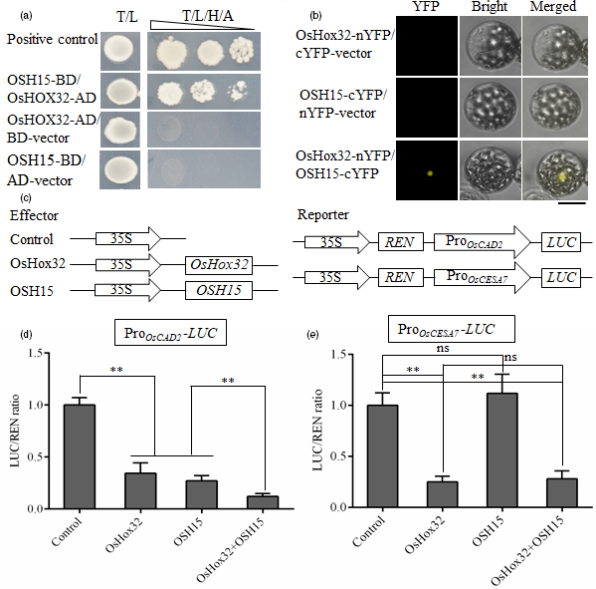
OsHox32 interacts with OSH15. (a) Y2H assay shows that OsHox32 interacts with OSH15. T/L, synthetic complete medium lacking Trp and Leu. T/L/H/A, synthetic complete medium lacking Trp, Leu, His and Ade. Triangle indicates 10‐fold dilution of yeasts. Positive control, pGADT7‐7/pGBKT7‐53 combination from Clontech Kit. (b) BiFC confirms the interaction of OsHox32 and OSH15. Bar = 20 μm. (c) Diagrams of the effector and the reporter constructs used in dual‐luciferase assay. (d, e) Dual‐luciferase assay shows that OsHox32 and OSH15 synergistically suppress the expression of *OsCAD2* (d), but the coexistence of these two proteins does not have the synergistic effect on the suppression of *OsCESA7* (e). Three biological repeats were performed. Student’s *t*‐test. **, *P* < 0.01, ns, not significant.

OSH15, a Class I KNOX protein, was reported to bind to *OsCAD2* promoter and suppress the expression of *OsCAD2*, leading to reduced lignin content (Yoon *et al.,* 2017). Yeast two‐hybrid (Y2H) assay showed that OsHox32 interacts with OSH15 (Figure 10a), and bimolecular fluorescence complementation (BiFC) experiment further confirmed the interaction between OsHox32 and OSH15 (Figure 10b). OsHox32 and OSH15 each contain several domains (Figure S5a), and we made the truncated proteins containing different domains for each protein. The Y2H assay showed that the START domain‐containing fragment of OsHox32 interacts with the KNOX box‐containing fragment of OSH15 (Figure S5b). On the other hand, OsHox32 binds to P4 and P5 parts of the *OsCAD2* promoter, while OSH15 binds to P5 and P6 parts of the *OsCAD2* promoter (Yoon *et al.,* 2017), and the overlap of the binding sites implies the effects of interaction on the gene expression. To investigate the regulatory effects of OsHox32 and OSH15 on the expression of *OsCAD2* and *OsCESA7*, we carried out the dual‐luciferase assay which showed that coexistence of OsHox32 and OSH15 synergistically suppressed the expression of *OsCAD2* (Figure 10c, d), but the coexistence did not affect the suppression of OsHox32 on the expression of *OsCESA7* (Figure 10c, e).

## Discussion

MicroRNAs play vital roles in many aspects of plant growth and development by mediating target gene expression. A single miRNA was predicted to target multiple genes, and the best way to elucidate the function of a miRNA is to find out its authentic targets and study the function of the pair of miRNA and target gene. miR166 is conserved in dicots and monocots and forms a big family of 24 members in rice (*Oryza sativa* L.) (http://structuralbiology.cau.edu.cn/PNRD/). OsmiR166b was reported to be located in a yield‐related QTL interval and differentially expressed in different tissues of an elite hybrid rice (Fang *et al.,* 2013), and it was confirmed to target *OsHox32*, a HD‐ZIP III family gene. Phenotypic observation of the OsmiR166b and *OsHox32* transgenic rice plants demonstrated that the OsmiR166b‐*OsHox32* pair regulates plant growth and development and architecture formation.

Zhang et al (2018) reported that knockdown of rice miR166 causes altered stem xylem development and decreased hydraulic conductivity, which leads to rolled leaf and thus confers drought resistance. Li et al (2016) found that overexpression of *OsHox32* results in narrow leaves and reduced plant height. In this study, we observed rolled leaves and reduced plant height in STTM166b and OEHox32 transgenic plants, which is in agreement with their findings. However, we also observed that STTM166b and OEHox32 transgenic plants displayed drooping leaf and brittle culms, which implies that the OsmiR166b‐*OsHox32* module plays important roles not only in controlling growth and development but also in modulating the mechanical strength of plants.

Mechanical strength of plants is determined by the components of cell walls. SEM and TEM analyses demonstrated that the thickness of cell walls of OsmiR166b and OsHox32 transgenic plants was different to that of WT. Mixed‐linkage glucan (MLG) is a polysaccharide and deposited in the primary cell wall to regulate cell wall expansion of the young and expanding tissues, and *CSLF6* has a predominant and nonredundant role in the biosynthesis and accumulation of MLG (Kim *et al.,* 2018; Vega‐Sánchez *et al.,* 2012). The *cslf6* knockout rice mutant not only showed a decrease in both plant height and stem diameter but also exhibited a significant reduction in culm strength (Vega‐Sánchez *et al.,* 2012). Cellulose biosynthesis is controlled by a set of CESA genes, and mutations in these genes result in changes in cellulose content and defects in plant growth and development. *OsCESA6*, partially redundant to *OsCESA3*, is responsible for cellulose biosynthesis in the primary cell wall (Wang *et al.,* 2010). Previous studies showed that cellulose deficiency in primary cell wall is correlated with cell elongation defects and morphology changes (Paredez *et al.,* 2006, Park and Cosgrove, 2012). The reductions in plant height and mechanical strength were also observed in OsmiR166b and OsHox32 transgenic plants, and these phenotypic changes may be partially due to the different expression of *OsCESA6* and *OsCSLF6*. Calcofluor staining assay showed much more cellulose deposition in OE166b and RNAiHox32 transgenic plants but less in STTM166b and OEHox32 plants. *CESA4* and *7* are required for cellulose biosynthesis during the secondary cell wall formation. Although the expression of *OsCESA4* was not significantly different between the wild‐type and transgenic plants, the expression level of *OsCESA7* was significantly regulated by OsmiR166b‐*OsHox32* pair, and molecular analyses further confirmed that OsHox32 binds to the promoter region of *OsCESA7* and suppresses its expression. The regulation of *OsCESA7* may lead to differential deposition of cellulose in the secondary cell walls of the transgenic plants.

In the generally accepted model for secondary cell wall, lignin is proposed to give the wall rigidity and resistance to compressive force (Turner and Somerville, 1997). Lignin biosynthesis genes and pathways have been uncovered and well defined (Raes *et al.,* 2003). CADs catalyse the last step of lignin biosynthesis, and among the 12 CAD genes present in the rice genome, *OsCAD2* is the major one responsible for monolignol biosynthesis in rice culm (Hirano *et al.,* 2012). *OsCAD2‐*mutant *gh2* showed reddish‐brown panicles and internodes and exhibited a decrease in lignin content in cell walls when compared to wild‐type plants (Zhang *et al.,* 2006). Besides the gold hull and internode, the *oscad2* rice mutant showed brown midrib and decreased lignin content as well (Koshiba *et al.,* 2013). In addition, the maize (*Zea mays* L.) *bm1* (*brown midrib 1*) mutant harbours mutations in the *CAD* gene which leads to a decrease in CAD activity and a reduction in lignin content (Halpin *et al.,* 1998). On the other hand, the maize *bm3* mutants showed structural changes in *COMT* gene and exhibited only 10% COMT activity of the WT (Vignols *et al.,* 1995). For lignin biosynthesis, *PAL1*, *C4H* and *4CL3* are responsible for the first three steps of the biosynthesis pathway, whereas *CAD* and *COMT* account for the rear steps of the pathway (Li *et al.,* 2015; Zhao, 2016). The reduced CAD or COMT activity found in either rice *gh2* mutant or maize *bm* mutants may feedback to the upstream steps of the biosynthesis pathway to stimulate the expression of genes such as *4CL3*, leading to the flux of intermediate products to the flavonoid biosynthesis branch that increases flavonoid contents and results in pigment formation in tissues. In this study, OsmiR166b‐*OsHox32* pair significantly down‐regulated the expression of *OsCAD2* and *Os4CL3* in STTM166b and OEHox32 plants, which slows down the biosynthesis speed of the whole lignin pathway and does not mediate the flux of intermediate products to flavonoid biosynthesis, therefore leading to less lignin accumulation and no pigment formation in the midribs and internodes of STTM166b and OEHox32 plants.

The OsmiR166b‐*OsHox32* pair regulates lignin biosynthesis through the binding of OsHox32 to the promoter of *OsCAD2*. Generally, the lignin biosynthetic genes are regulated by a pyramid‐shaped regulatory hierarchy of transcription factors (Nakano *et al.,* 2015; Zhao and Dixon, 2011). NAC and MYB transcription factors have been demonstrated to act as master switches of the entire secondary cell wall biosynthesis. SND1 is the first‐layer master switch for secondary cell wall biosynthesis and controls the second‐layer master switches of *MYB46* and *MYB83* by binding to their promoters (McCarthy *et al.,* 2009). Except for this general transcriptional cascade, other ways are also found to be involved in the secondary cell wall biosynthesis. For example, lignin laccase genes were demonstrated to be the targets of miR397, and the miR397‐mediated cleavage of laccase transcripts leads to reduced lignin content (Lu *et al.,* 2013; Wang *et al.,* 2014). *OSH15* is a member of the *knotted1*‐type homeobox gene family, and mutations in this gene caused a dwarf phenotype and an increased deposition of lignin in internodes (Sato *et al.,* 1999; Yoon *et al.,* 2017). Molecular investigation showed that OSH15 is a negative regulator of lignin biosynthesis as it binds to the promoter region of *OsCAD2* and suppresses the expression (Yoon *et al.,* 2017). OsHox32 acts as another negative regulator of *OsCAD2* by binding to the promoter and suppressing the expression of *OsCAD2*. In this case, *OsCAD2* is regulated by two negative factors, and the binding sites for these two proteins, OsHox32 and OSH15, are overlapped on the promoter region. OsHox32 interacts with OSH15, and the interaction enhances the suppression of *OsCAD2*. Since *OsCAD2* is the *bona fide* CAD gene that plays important roles in monolignol biosynthesis (Hirano *et al.,* 2012), regulation of *OsCAD2* expression may act as the key switch to control lignin content. Overexpression of *OsHox32* not only increases suppression of *OsCAD2* by OsHox32 itself but also enhances the suppression of *OsCAD2* by OSH15, thus greatly reduces lignin content in tissues. However, lignin is essential for plant growth and development, and sometimes suppression of *OsCAD2* should be released or controlled to some extent. In this respect, OsmiR166b exerts power to affect lignin biosynthesis by cleaving *OsHox32* transcript and releasing the OsHox32 suppression on *OsCAD2*. That the OsmiR166b‐*OsHox32* pair regulates lignin biosynthesis represents an elaborate way that is more efficient than the direct way exemplified by miR397 and OSH15‐mediated regulation. The diversity of the regulatory pathways might be dominant in a context‐dependent manner, but how these pathways corporately regulate lignin biosynthesis needs further investigation.

## Methods

### Plant materials and growth conditions

All transgenic plants were generated from rice (*Oryza sativa* L.) Zhonghua 11 (*japonica* cv. ZH11). Multiple transgenic plants were obtained, and two representative homozygous lines for each gene transformation were selected to use in this study. Rice plants including transgenic lines and wild‐type ZH11 plants were grown in a paddy field under natural conditions during summer and autumn seasons at South China Botanical Garden, Guangzhou, China, and treated with normal management.

### Plasmid constructs and rice transformation

To specifically block the function of OsmiR166b, we applied the short tandem target mimic (STTM) method (Yan *et al.,* 2012), in which the STTM module contained two copies of OsmiR166b that were linked by a 48 nt spacer. Overexpression of miR166b (OE166b) was constructed by inserting the PCR‐amplified miR166b precursor fragment into pCAMBIA1301 vector and expressed under control of the 35S promoter.

The full‐length cDNA fragment of *OsHox32 w*as amplified and inserted into pCAMBIA1301 vector and expressed under control of the 35S promoter. The resulting construct was used to overexpress *OsHox32* in rice plants. To generate *OsHox32*‐RNAi transgenic plants, we PCR‐amplified a 214 bp fragment specific to the first exon of the *OsHox32* gene and inserted it into pTCK303 vector in opposite directions. The resulting construct was used to suppress the expression of *OsHox32* in rice plants.

All constructs were individually transformed into *Agrobacterium tumefacines* strain EHA105, and the transformed EHA105 strains were used to transform rice calli to generate transgenic plants. Totally, we got 11, 18, 17 and 15 transgenic lines for STTM166b, OE166b, RNAiHox32 and OEHox32, respectively. The primer sequences used for plasmid construction are listed in supporting information Table S2.

### Gene expression analysis

Total RNAs were extracted from leaves collected from plants at tillering stage, and the first‐strand cDNAs were generated from equal amounts of total RNAs using a reverse transcription kit (TaKaRa, Shiga, Japan). Quantitative reverse transcription PCR was performed with three biological repeats on a Roche Light Cycler 480 real‐time PCR machine with SYBR Premix ExTaq II (TaKaRa). PCRs were carried at 95 °C for 30 s followed by 40 cycles of 95 °C for 10 s, and 60 °C for 10 s, and then 72 °C for 15 s. The rice *Actin1* gene was used as the internal control, and relative expression levels of genes were calculated using the 2^−∆∆CT^ method (Livak and Schmittgen, 2001). Primer sequences are listed in Table S2.

### Protein–protein interaction analysis

For yeast two‐hybrid (Y2H) analysis, the full coding sequence fragment of *OsHox32* or *OSH15* was accordingly cloned into the pGBKT7 or pGADT7 vector. In addition, the truncated *OsHox32* fragments containing either the HD domain or the START domain were accordingly cloned into the above vectors as well. On the other hand, the truncated *OSH15* fragments containing either the KNOX domain or the ELK or the HD domain were cloned into one of the above vectors. Various combinations of different constructs were tested for protein–protein interaction in yeasts. The Y2H assay was performed following the manufacturer’s instructions (Clontech, Palo Alto, CA).

For bimolecular fluorescence complementation (BiFC) analysis, the full‐length fragment of the *OsHox32* (without stop codon) coding region was cloned into pUC‐SPYNE to generate OsHox32‐nYFP fusion protein. On the other hand, the full‐length fragment of the *OSH15* coding region (without stop codon) was cloned into pUC‐SPYCE vector to generate OSH15‐cYFP fusion protein. These two constructs were combined to transfect Arabidopsis protoplast cells, and the cells were then incubated at 23 °C for 16 h. Yellow fluorescent protein (YFP) fluorescence was checked by DMI6000B inverted fluorescence microscope (Leica, Wetzlar, Germany) under DAF channel. Primer sequences are listed in Table S2.

### ChIP‐PCR assay

Plasmid DNAs of OsHox32‐3flag and pCactF‐3flag were used to transfect rice protoplast cells (Zhang *et al.,* 2011). The transfected cells were incubated at 26°C for 12 h and then cross‐linked with 1% formaldehyde solution (in 1× PBS buffer with pH 7.4). Chromatins were isolated and sheared to an average length of 500 bp by sonication. The solubilized chromatins were immunoprecipitated with anti‐flag antibody. The cross‐linking was then reversed, and the co‐immunoprecipitated DNAs were then recovered and analysed by quantitative PCR with SYBR Premix ExTaq II (TaKaRa) using gene‐specific primers. ChIP assays were performed as described previously (Lee *et al.,* 2017). Sequences of the primers are listed in Table S2

### Yeast one‐hybrid assay

For yeast one‐hybrid (Y1H) analysis, the 146 bp fragment of the *OsCAD2* promoter region (from −593 to −447 upstream of the start codon) and 119 bp fragment of the *OsCESA7* promoter region (from −1674 to −1555 upstream of the start codon) were PCR‐amplified and inserted into the pAbAi vector, respectively. The full‐length cDNA of *OsHox32* was cloned into pGADT7 vector. Yeast transformation and assay were performed following the manufacturer’s instructions (Clontech, Mountain View, CA). Primer sequences are listed in Table S2.

### Dual‐luciferase assay

For dual‐luciferase reporter assays, the coding sequences of *OsHox32* and *OSH15* were individually PCR‐amplified and cloned into the pGreenII 62‐SK vector as effectors, whereas the promoter region of *OsCAD2* (−2500 bp upstream of ATG), were inserted into pGreenII 0800‐LUC vector as a reporter. The reporter and effectors were co‐transfected into Arabidopsis protoplast cells in different combinations and incubated at 23 °C for 12 h. Firefly LUC and REN activities were measured by a multimode reader (infinite M200PRO) using the Dual‐Luciferase Reporter Assay System (Promega, Fitchburg, WI, USA). The parameter used for measurement was chemiluminescence, and integration time was 5000 ms. Three biological repeats were performed.

### Scanning electron microscopy

The central parts of the second internodes from the top of rice plants at heading stage were collected and excised into small pieces with a razor, and the pieces were immediately placed in the solution of 70% ethanol, 5% acetic acid and 3.7% formaldehyde for 18 h. Samples were critical point dried, sputter‐coated with gold in an E‐100 ion sputter (Mito City, Japan) and observed with a scanning electron microscope (JSM‐6360LV, Japan).

### Transmission electron microscopy

The central parts of flag leaf blade at tillering stage were harvested and excised into small pieces, which were then fixed in the solution of 2.5% (w/v) glutaraldehyde in 0.1 m PBS (4 mm sodium phosphate, pH 7.2; 200 mm NaCl) at 4 °C for overnight. Samples were dehydrated through a gradient of ethanol and embedded with Spur Kit (Sigma, Darmstadt, Germany). The 80‐nm ultrathin sections were prepared with an Ultracut E ultramicrotome (Leica) and picked up on formvar‐coated copper grids. After post‐staining with uranyl acetate and lead citrate, the specimens were observed under a transmission electron microscope (JEM‐1010, Japan).

### Cellulose and lignin histochemical staining

For cellulose staining, paraffin‐embedded transverse sections (20 μm thickness) of the central parts of the flag leaf blade at tillering stage and of the central parts of the second culms at heading stage were stained with a drop of Calcofluor White Stain (Sigma, Darmstadt, Germany) and a drop of 10% KOH for 1 min and then visualized with a fluorescent microscope (Leica). Images were captured GFP excitation, a range of 375–525 nm.

For histochemical localization of lignin, materials from the same parts of leaves and culms at the same stage as those used for cellulose staining were used. Paraffin‐embedded transverse sections (20 μm thickness) of leaves and culms were stain with phloroglucinol‐HCl solution for 2 min in phloroglucinol solution (5% in ethanol: water [95:5, v/v]; 36% HCl) to distinguish the lignified cell walls (Anderson *et al.,* 2015), and then, sections were photographed using LEICA DVM6 stereomicroscope.

### Cellulose and lignin measurement

To measure cellulose and lignin contents, flag leaves of rice plants at tillering stage and the second culms from the top of rice plants at heading stage were harvested, respectively, and then prepared into alcohol‐insoluble residue (AIR) (Harholt *et al.,* 2006). Tissues were ground into fine power in liquid nitrogen, dissolved with 80% ethyl alcohol and then boiled at 100 °C for 20 min. After cooling to room temperature, samples were centrifuged at 2000 g for 10 min and the supernatant was removed. The residues were resuspended in 80% ethyl alcohol, then in 80% acetone, and then in 100% acetone, and finally air‐dried. The air‐dried residues were treated with pullulanase M3 (0.5 U/mg, Megazyme) and alpha‐amylase (0.75 U/mg, Sigma) in 0.1 m NaOAc buffer (pH 5.0) for overnight, and then centrifuged at 2000 g for 10 min, the residues were washed with 80% ethyl alcohol and then dried at 35°C for overnight to get AIR.

The cellulose content was measured using a modified method as described by Updegraff (1969). In brief, 100 mg AIR were hydrolysed with 3 mL Updegraff reagent (Acetic acid: HNO_3_ : ddH_2_O = 8:1:2) by boiling at 100°C in a water bath for 30 min and then cooled to room temperature. After centrifugation at 2000 g for 10 min, the supernatant was removed, and the residues were washed with distilled water once and then hydrolysed with 67% sulphuric acid for 1 h. Samples were then centrifuged at 2000 g for 10 min. Use 1 mL supernatant and dilute to 100 mL with distilled water. Then adding 10 mL ice‐cold anthrone reagent to solution and then boiling at 100 °C in a water bath for 15 min. After that, samples were cooled on ice for 2 min and then left at room temperature for 10 min. Finally, samples were read at 620 nm using a spectrophotometer (UV‐1800).

The lignin content was quantified according to the method described by Fukushima and Hatfield (2001). Briefly, 100 mg AIR were hydrolysed with 4 ml 25% acetyl bromide at 50 °C in a water bath for 2 h, then adding acetic acid to bring the volume to 16 mL. Then mixed 0.5 mL reaction solution with 7.5 mL acetic acid, 1.5 mL 0.3 m NaOH and 0.5 mL 0.5 m hydroxylamine hydrochloride at room temperature for 30 min. Samples were read at 280 nm using a spectrophotometer (UV‐1800).

## Conflict of interests

The authors declare no competing interests.

## Authors’ contributions

H.C. performed most experiments, including vector constructions, PCR, protein–protein interaction, ChIP‐PCR, dual‐luciferase assay, histochemical staining, cellulose and lignin content measurement, R.Q.F. completed STTM construction and obtained STTM166b transgenic plants. R.F.D. helped in SEM and TEM. J.X.L. conceived and supervised the project. H.C. and J.X.L. analysed data and wrote the manuscript.

## Supporting information


**Figure S1** OsmiR166b targets Os*Hox32*

**Figure S2** Characterization of transgenic plants

**Figure S3** Comparison of the internodes and leaves from ZH11 and transgenic plants

**Figure S4** Hemicellulose content of the flag leaves from wild‐type and transgenic plants at tillering stage

**Figure S5** Interaction between the truncated proteins of OsHox32 and OSH15

**Table S1** Agronomic trait investigation for the OsmiR166b and OsHox32 transgenic rice plants.

**Table S2** Sequences of primers used in this study.
