# Supplementary figures and images for: The OsmiRNA166b‐OsHox32 pair regulates mechanical strength of rice plants by modulating cell wall biosynthesis
Source: Plant Biotechnol J. 2021 Mar 5;19(7):1468–80. doi: 10.1111/pbi.13565 (PMC8313131; doi:10.1111/pbi.13565)

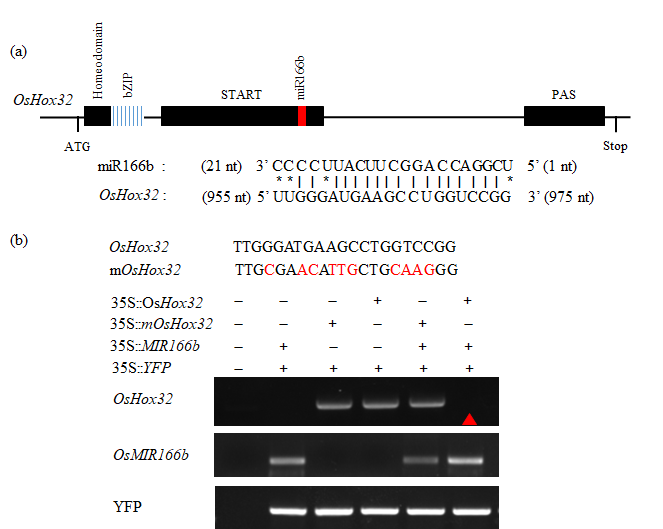

Supplement: Supplementary file 1 — Figure S1 OsmiR166b targets OsHox32 [file PBI-19-1468-s003.tif]

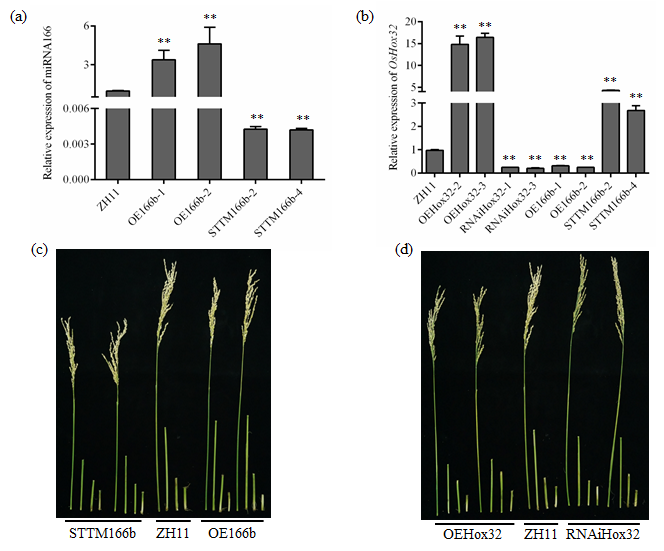

Supplement: Supplementary file 2 — Figure S2 Characterization of transgenic plants [file PBI-19-1468-s001.tif]

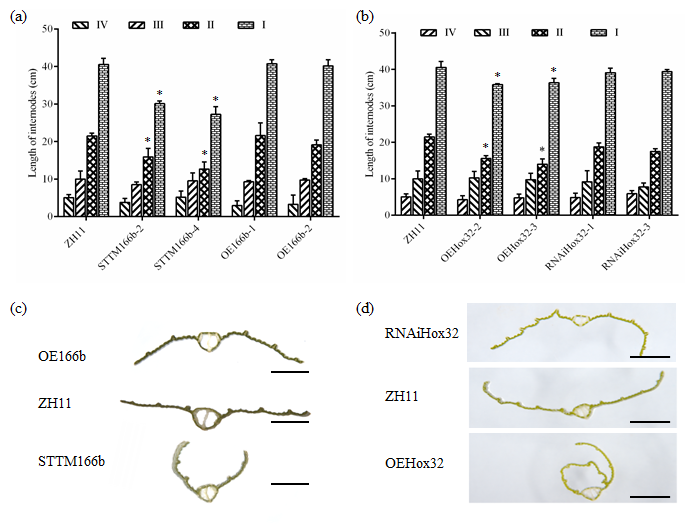

Supplement: Supplementary file 3 — Figure S3 Comparison of the internodes and leaves from ZH11 and transgenic plants [file PBI-19-1468-s002.tif]

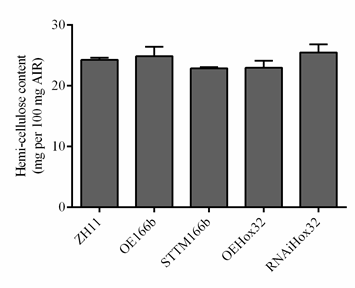

Supplement: Supplementary file 4 — Figure S4 Hemicellulose content of the flag leaves from wild‐type and transgenic plants at tillering stage [file PBI-19-1468-s005.tif]

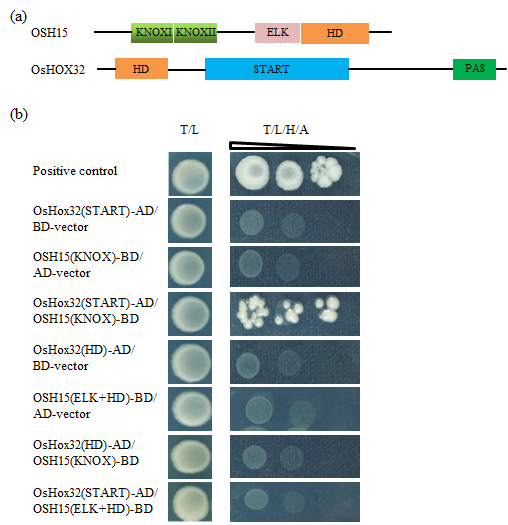

Supplement: Supplementary file 5 — Figure S5 Interaction between the truncated proteins of OsHox32 and OSH15 [file PBI-19-1468-s004.tif]
