# Supplementary material for: The OsmiRNA166b‐OsHox32 pair regulates mechanical strength of rice plants by modulating cell wall biosynthesis
Source: Plant Biotechnol J. 2021 Mar 5;19(7):1468–80. doi: 10.1111/pbi.13565 (PMC8313131; doi:10.1111/pbi.13565)
Supplement: Supplementary file 6 — Table S1 Agronomic trait investigation for the OsmiR166b and OsHox32 transgenic rice plants. [file PBI-19-1468-s006.docx]

Table S1. Agronomic trait investigation for the OsmiR166B and OsHox32 transgenic rice plants

| Trait | ZH11 | OE166b | STTM166b | OEHox32 | RNAiHox32 |
| --- | --- | --- | --- | --- | --- |
| 1000-grain weight (g) | 24.38±0.65 | 24.50±0.60 | 18.60±0.44** | 22.90±0.47** | 24.88±0.87 |
| Panicles/Plant | 11.00±1.35 | 10.00±1.26 | 7.00±1.13** | 6.50±1.51** | 9.70±0.90 |
| Plant height | 98.92±5.71 | 99.81±5.30 | 83.07±5.22** | 89.30±6.358* | 98.75±2.71 |
| Grains/Plant | 179.91±8.74 | 188.36±7.24 | 115.18±6.32** | 157.82±4.12* | 192.18±8.85 |
| Spikelet fertility (%) | 89.86±1.07 | 85.59±1.19 | 67.40±3.08** | 69.32±2.62** | 83.32±1.15 |

Data in the same row were analyzed for each trait, and the significance was indicated as *, p < 0.05; **, p <0.01 0.01
