# Supplementary material for: The OsmiRNA166b‐OsHox32 pair regulates mechanical strength of rice plants by modulating cell wall biosynthesis
Source: Plant Biotechnol J. 2021 Mar 5;19(7):1468–80. doi: 10.1111/pbi.13565 (PMC8313131; doi:10.1111/pbi.13565)
Supplement: Supplementary file 7 — Table S2 Sequences of primers used in this study. [file PBI-19-1468-s007.docx]

**Table S2.** Sequences of primers used in this study

| **Primer name** | **Forward primer** | **Reverse primer** |
| --- | --- | --- |
| STTM166b | GCGCGTCGGACCAGGCTTCA | GTCGTATCCAGTGCAGGGTCCGAGGTATTCGCACTGGATACGACGGGGAA |
| U6-q-F | TACAGATAAGATTAGCATGGCCCC | GGACCATTTCTCGATTTGTACGTG |
| mHox32 | CCCTTGCAGCAAGGTTCGCAACCATTTGCAC | TGCGAACCTTGCTGCAAGGGATTCCATTGGAA |
| HOX32-HOX-F | GAAGAATTCATGGACACGGGCAAGTACGTGC | CTTGGATCCTCAAGATGCCTCCTTGCGCTGC |
| HOX32-START | GAAGAATTCATGGCTATAGCTGAGGAGACC | CTTGGATCCTCAAATTTGCCTAATGTGACGCAG |
| OSH15-KNOX | GAAGAATTCATGGAGTCCATCAAGGCCAAGATC | CTTGGATCCTCAGCCGGCGATGGTGTCGAG |
| OSH15-HOX | GAAGAATTCATGGAGCTCAAGTTTCAGCTTCTG | CTTGGATCCTCACATGTCCTCCGATGGCTTC |
| OsHox32-RNAi | GGAGGTACCACTAGTCCTCCTCCTCCTCCTCACCACC | GTTGGATCCGAGCTCCCCGTGTCCACTTGCGGCGC |
| OsHox32-OE | ATTGAGCTCATGGCGGCGGCGATGGTG | ATATCTAGATCAGACGAATGACCAGTTGACGAAC |
| flag-HOX32 | GTTTCTAGAATGGCGGCGGCGATGGTG | GTTGTCGACTCAGACGAATGACCAGTTGACGA |
| CAD2-ChIP-1 | ACAAGGACATGATGGCTACTG | GCGACTTTAGTTAGTAGCTTGA |
| CAD2-ChIP-2 | GGGAATCTGTTAGGGTTATGGA | TATACCCATAGGTGTCCCCTTG |
| CAD2-ChIP-3 | GGTTGTAGGGCTATTACCTGAC | CTCGGTTATTACGTTCACTATCC |
| CAD2-ChIP-4 | GGATAGTGAACGTAATAACCGAG | CTATTCAACTACTTTATCCGTATC |
| CAD2-ChIP-5 | GATACGGATAAAGTAGTTGAATAG | GTTAGTAGTAGGACAAGTAGTG |
| CAD2-ChIP-6 | CTCAGTCACGACCCGTCCAAC | CCAATGGATGGATCGGATTC |
| CAD2-ChIP-7 | CAACTACACCCTCAGGTATGAT | CTAAACTGTAGTAGAACGCATGT |
| CESA7-ChIP-1 | TTGCTTCAGCTGCACTACGA | GGCGTCCCGGTTTGATTAGA |
| CESA7-ChIP-2 | TCCTCCTACCGTCTGCCATT | GGCGTCCCGGTTTGATTAGA |
| CESA7-ChIP-3 | TCACTGCACACCTGATTCGT | GTGTTGGAGGGGTCGTAGTG |
| CESA7-ChIP-4 | CATCGAGTGGTGTGCCGATA | TTCAGGGAGGCTCTGGTGTA |
| CESA7-ChIP-5 | CATGCCTTTGCTTTGCAGGT | TGCCATGGCTCCTCTTGATT |
| CESA7-ChIP-6 | GGTTTGCATTGATGAGCACCAT | AGCACCATCCAAATAGAATGAGC |
| CESA7-ChIP-7 | TGCCGTGTCAAAGAGACTCAA | TGTCCTTTGAATGCACAGACCT |
| CESA7-ChIP-8 | TTTGTTTGCTTTTCTGAGTTCCA | GCTCATCAATGCAAACCAGC |
| CESA7-ChIP-9 | ACATTGTTTTCCCACCACCTCT | AGCCTACCATCAAATACTTGTCA |
| CAD2-Pro | GTTAAGCTTCCCGGTTAGTCCACTCAGCC | CTTGGATCCTGTGGAGCCAGGCAACTAGC |
| HOX32-Effector | GTTGGATCCATGGCGGCGGCGATGGTG | GTTGAATTCTCAGACGAATGACCAGTTGACGA |
| OSH15-Effector | CCCGGATCCATGGATCAGAGCTTTGGGAA | TTTGAATTCTCACGAACCGAGGCGGTACA |
| HOX32-Y2H | GTTGAATTCATGGCGGCGGCGATGGTG | GTTGAATTCTCAGACGAATGACCAGTTGACGA |
| HOX32-BIFC | GTTGAATTCATGGCGGCGGCGATGGTG | GTTGTCGACGACGAATGACCAGTTGACGA |
| OSH15-Y2H | CCCGAATTCATGGATCAGAGCTTTGGGAA | TTTGGATCCTCACGAACCGAGGCGGTACATTC |
| OSH15-BiFC | CCCGGATCCATGGATCAGAGCTTTGGGAA | TTTCTCGAGCGAACCGAGGCGGTACATTC |
| OsCSLF6-q | GGTGTGGTGTATGATTGT | CAGCCTCTCTTTCTCTCA |
| OsCESA6-q | GAGCTTACTTGGTGGAAG | AATAAACAATTGGCATCTTC |
| Os4CL3-q | ATCTTGCCGGTGAAGTGCCA | TGCAACGAATTTCTTGATCT |
| OsCOMT-q | CCTCGCCCTCATGAACCA | CGTCCAGGACTGCGTCCTTA |
| OsCESA7-q | TCCATCTTCTCCCTCGTCTG | GAATCATCCATCCGGTCATC |
| OsPAL1-q | AGGAGCTCGGCTGCGTATT | ATGCCGAGGAACACCTTGTT |
| OsCAD2-q | CTGAAGATGTGGTGGTGAAG | GACCATGGGGTACTTGGAAG |
| OsCesA4-q | CTAATGCGACGAAGACGATG | GATTTAACGGTGCCCTCTCA |
| OsHox32-q | GCGGCAGCAGCTCCATAATC | TAGACCAGCTGGGTTGTTCG |
| OsActin1-q | CTCCCCCATGCTATCCTTCG | TGAATGAGTAACCACGCTCCG |
